# Computationally-defined markers of uncertainty aversion predict emotional responses during a global pandemic

**DOI:** 10.1037/emo0001088

**Published:** 2022-06-06

**Authors:** Toby Wise, Tomislav D Zbozinek, Caroline J. Charpentier, Giorgia Michelini, Cindy C Hagan, Dean Mobbs

**Affiliations:** 1 Division of Humanities and Social Sciences, California Institute of Technology, Pasadena, CA; 2 Department of Neuroimaging, Institute of Psychiatry, Psychology and Neuroscience, King’s College London, London, UK; 3 Department of Psychology, Queen Mary University of London, London UK; 4 Semel Institute for Neuroscience and Human Behavior, University of California Los Angeles, Los Angeles, CA; 5 Institute of Cognitive Neuroscience, University College London, London, UK; 6 Computational Neural Systems Program, California Institute of Technology, Pasadena, CA

## Abstract

Exposure to stressful life events involving threat and uncertainty often results in the development of anxiety. However, the factors that confer risk and resilience for anxiety following real world stress at a computational level remain unclear. We identified core components of uncertainty aversion moderating response to stress posed by the COVID-19 pandemic derived from computational modelling of decision making. Using both cross-sectional and longitudinal analyses, we investigated both immediate effects at the onset of the stressor, as well as medium-term changes in response to persistent stress. 479 subjects based in the United States completed a decision-making task measuring risk aversion, loss aversion, and ambiguity aversion in the early stages of the pandemic (March 2020). Self-report measures targeting threat perception, anxiety, and avoidant behavior in response to the pandemic were collected at the same time point and 8 weeks later (May 2020). Cross-sectional analyses indicated that higher risk aversion predicted higher perceived threat from the pandemic, and ambiguity aversion for guaranteed gains predicted perceived threat and pandemic-related anxiety. In longitudinal analyses, ambiguity aversion for guaranteed gains predicted greater increases in perceived infection likelihood. Together, these results suggest that individuals who have a low-level aversion towards uncertainty show stronger negative emotional reactions to both the onset and persistence of real-life stress.

## Introduction

Stressful life events can have dramatically different emotional effects across individuals. While some respond to such events with little emotional distress, life stress can result in severe negative psychological effects for many (Bonanno & Mancini, 2012; Kessler, 1997), with anxiety being a common result of stress (Blazer et al., 1987; Kendler et al., 2003). However, the source of this heterogeneity remains unclear, largely due to the challenges inherent in measuring responses to salient real-world stressful life events. Here, we tested whether uncertainty aversion moderated responses to the COVID-19 pandemic, a real world stressor on a scale rarely experienced by most individuals.

While laboratory investigations of stress have examined this individual variation, they are also limited by ethical constraints and are often phasic in their administration (for example using electric shocks (Robinson et al., 2013), or stress inductions such as Trier social stress test (Kirschbaum et al., 1993). Outside the lab, existing work has identified effects of psychological factors including positive affect (Sewart et al., 2019) and memory biases (Sumner et al., 2011) in moderating the relationship between life stress and anxiety; however this work has limitations. First, these studies typically rely on measures of participant-reported naturally occurring stress, which is vulnerable to biases in subjects’ perception of stressful events(Dohrenwend, 2006). Second, naturally occurring stress may be confounded with variables of interest, for example genetic factors may influence both life stress and anxiety (Plomin, 2013). Finally, while existing work has evaluated psychosocial factors mediating responses to stress extensively, more modern, mechanistic approaches focusing on computational models of behavior are gaining traction within psychology and psychiatry (Friston et al., 2014; Montague et al., 2012), but have yet to be tested in the context of life stress. In particular, no studies have investigated whether affective decision-making styles affect responses to life stress.

Candidate moderating factors include biases in how people respond to uncertainty. Anxiety is associated with intolerance of uncertainty (Carleton et al., 2012) and risk aversion (Charpentier et al., 2017; Harpaz Rotem et al., 2017; Lerner & Keltner, 2000, 2001; Maner et al., 2007; Raghunathan & Pham, 1999) (i.e., the tendency to prefer more certain options over uncertain options (Abdellaoui et al., 2008, 2016; Kahneman et al., 1991; Kahneman & Tversky, 2013; Tversky & Kahneman, 1992)). In addition to disliking uncertainty in the *likelihood* of outcomes occurring, anxious individuals may also dislike uncertainty in the *magnitudes* of those outcomes. For example, clinical studies suggest that anxiety increases predictions of the likelihood and magnitude of potential negative events (Amir et al., 2005; Butler & Mathews, 1983; Eisenberg, A. E. et al., 1998; Lerner & Keltner, 2001). Indeed, clinical anxiety is associated with catastrophizing (Beck et al., 1974), which involves overestimating how severe a negative outcome will be or underestimating how good a positive outcome will be. Furthermore, anxiety is consistently associated with increased negative affect (Brown et al., 1998; Prenoveau et al., 2010; Watson, 2005) and avoidance of objectively safe situations for fear of negative outcomes (Craske et al., 2012; Gazendam et al., 2013). Together, these findings suggest that individuals with clinical anxiety may overestimate the likelihood and magnitude of negative outcomes, especially if the likelihood and magnitude are uncertain. These biases may play a role in the way individuals respond to stressful life events, particularly if there is a degree of uncertainty about the likelihood or severity of the event. In line with this hypothesis, intolerance of uncertainty has been found to moderate the link between stressful life events and anxiety (Chen & Hong, 2010; Oglesby et al., 2016).

Recent research in computational psychiatry has allowed these biases to be quantified with greater precision, facilitating inference about their role in psychopathology (Friston et al., 2014; Montague et al., 2012). Specifically, a number of previous studies have reported that processes involved in learning and decision making under uncertainty play a role in both trait and clinical anxiety (Aylward et al., 2019; Charpentier et al., 2017; Huang et al., 2017; Wise & Dolan, 2019). Specifically, three main aspects of decision making have been highlighted as playing important roles: risk aversion, loss aversion and ambiguity aversion. Risk aversion represents the tendency of an individual to avoid options that involve uncertain outcomes (in terms of outcome probability) relative to sure options. Loss aversion indicates the tendency to overweigh losses relative to gains of equivalent magnitude. Ambiguity aversion represents a tendency to prefer outcomes of known magnitude versus outcomes of unknown magnitude. Assessing these processes using carefully designed decision-making tasks and computational models can thus provide a formal account of the biases described in clinical anxiety. Yet, there is little research examining how individual differences in perceptions of outcome likelihood and magnitude relate to responses to real-world stressful life events.

**Figure 1:**
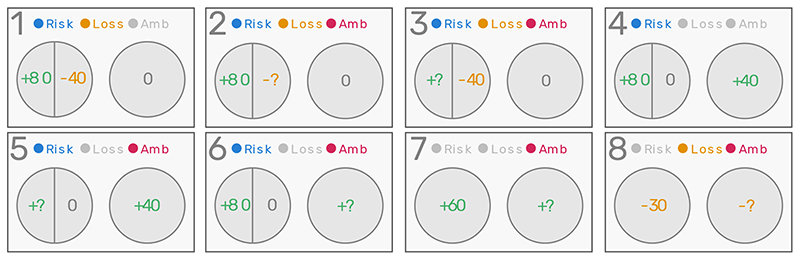
Task conditions, representing combinations of risk, loss, and ambiguity. On each trial, subjects chose between two options, each shown on the left and right of the screen within a circle. Each option could contain either a single “sure” outcome, or two “risky” outcomes. Outcomes could either be positive (+), negative (-) or zero, and could be unambiguous, where the potential outcome was shown to the subject (e.g. 40), or ambiguous, where the potential outcome was not shown (?).

In the current study we evaluated the extent to which loss aversion, risk aversion, and ambiguity aversion predicted emotional responses (perceived threat, behavior, and subjective anxiety) during the COVID-19 pandemic. The pandemic presented individuals globally with a multitude of stressors including economic uncertainty, uncertainty around health, and social isolation (Bavel et al., 2020) on a scale rarely seen. The onset of this tragic event, however, represented a rare opportunity to examine a sustained global stressor and answer an important question: how do affective decision making processes moderate real-world reactions to stress? We assessed decision making styles and affective responses in an online study of subjects based United States in the first week after COVID-19 was declared a pandemic by the World Health Organization (Figure 1)(World Health Organisation, 2020), when awareness was increasing but major effects were yet to be felt. Participants were re-assessed 8 weeks later, after substantial effects of the pandemic had hit the country. This allowed us to answer two questions: 1) how are decision making styles associated with immediate emotional reactions to a severe stressor at its onset? And 2) do decision making styles predict how these emotional reactions evolve over time? This was an exploratory study seeking to understand how different decision-making styles relate to emotional responses and suggest promising avenues for future research, and we did not set out to test any specific *a priori*-specified hypotheses.

## Methods

### Subjects

We recruited 479 subjects online using Prolific (Palan & Schitter, 2018). This was intended to allow the detection of a correlation of 0.2 with 80%, conservatively estimating 50% drop out over the two time points. Subjects completed the task and initial questionnaire battery on 3/14/2020 when awareness of COVID-19’s severity was rising in the United States, but before any government orders had been put in place restricting business activity and access to public spaces. As such, this provided a window of time where the future course of the pandemic was uncertain. We also recruited a further 1101 subjects who completed questionnaire measures between 3/11/2020 and 3/16/2020 but did not perform the gambling task at this time point; these participants were used for exploratory factor analysis (see below). 260 of the initial 479 subjects completed the same questionnaire measures 8 weeks later, in the week starting 5/4/2020. We included subjects who were based in the United States, were aged 18-65 years, and had at least a 95% approval rate on Prolific.

**Figure 2:**
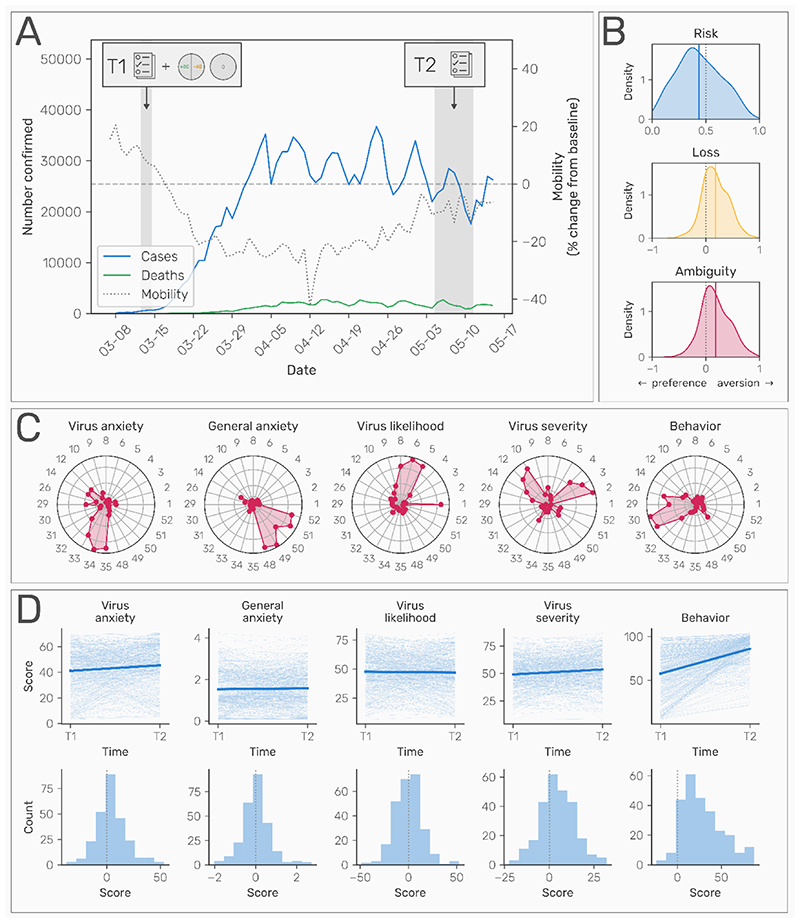
A) Study session in relation to the United States COVID-19 pandemic. At time 1 (T1), completed between 3/13/20 and 3/14/20, subjects completed questionnaire measures and the gambling task. At time 2 (T2), completed between 5/4/20 and 5/11/20, subjects completed only questionnaires. For reference, daily confirmed SARS-nCov-2 cases and deaths are shown over this time period, demonstrating the dramatic increase in the scale of the pandemic between the two time points, along with mobility data indicating the decrease in population-level movement over the time period relative to median mobility in January and February (data from https://www.google.com/covid19/mobility/). B) Distributions of risk aversion, loss aversion, and ambiguity aversion in the sample, as assessed by approximate behavioral indicators. Risk aversion is represented by the total number of gambles chosen, loss aversion as the contrast between condition 4 and condition 1, and ambiguity aversion as the contrast between condition 4 and 6. The solid vertical line indicates the mean across subjects. C) Factor loadings for the five factors identified using the questionnaire data. Numbers represent item numbers, and item descriptions are provided in Table S1. D) Top: Change in scores on the five factors identified through factor analysis of the questionnaire data between time 1 and time 2. Bottom: Distributions of latent change scores from longitudinal models.

These time points are of interest for a number of reasons (Figure 2A). The early time point in mid-March immediately followed the classification of the COVID-19 outbreak as a pandemic by the World Health Organisation (World Health Organisation, 2020), but recorded case numbers in the United States were low (2,234 total cases on 3/14/2020 (CDC, 2020b)), and population-level movement was largely unchanged from February (CDC, 2020a). As such, this marked a point where individuals in the United States were aware of the pandemic but largely unaffected. In contrast, at the start of the follow-up window there had been 1,171,510 total cases in the country (CDC, 2020a), but the number of new cases per day had begun to decrease (CDC, 2020b) after restrictions such as stay-at-home orders and business closures had been in place across many states, as evidenced by a reduction in population-level movement (CDC, 2020a). As such, these two time points represent moments before and after the effects of the pandemic had been felt by most United States residents.

### Questionnaires

Subjects completed a battery of questionnaires designed to measure attitudes towards the COVID-19 pandemic in addition to general anxiety in the past week, which was measured using the Overall Anxiety Severity and Impairment Scale (OASIS). Results from this full questionnaire battery have been reported elsewhere (Wise et al., 2020), and here we focus on a subset of the measures that targeted risk perception, behavior, and anxiety. These were chosen as we wished to focus on anxiety in response to the unfolding pandemic, covering perceived risk and behavioral components of this emotional response in addition to the subjective experience of anxiety. Specifically, we used 24 items relating to individual perceived infection likelihood and severity, alongside measures of virus-related anxiety and engagement in protective behaviors. Subjects responded to each item using a visual analogue scale ranging from “strongly disagree” to “strongly agree”. Prior to beginning this analysis, we excluded outlying subjects based on their multivariate distance from the sample distribution, determined using the Mahalanobis distance measure (De Maesschalck et al., 2000). Subjects were excluded if their distance score exceeded the *p* < .001 level of the *X*^2^ distribution.

### Factor analysis

To reduce the dimensionality of our self-report measures, we performed exploratory factor analysis using minimum residual estimation with an oblimin rotation, allowing us to identify latent factors underlying the various measures. This was implemented in R 3.6.1 using the Psych package. We determined the appropriate number of factors using the scree method, using the factors where the screen plot levelled. Exploratory factor analysis was performed at baseline in 1101 subjects who did not perform the task.

Following exploratory factor analysis, we used confirmatory factor analysis (CFA) to evaluate the fit of the factor model in the subjects who completed the task at baseline. We used a cutoff of 0.5 in determining factor loadings for the CFA model, which was fit using Lavaan in R 3.6.1. We evaluated model fit using the root mean squared error approximation (RMSEA), *X*^2^, comparative fit index (CFI) and standardized root mean square residual (SRMR). Finally, as we intended to assess change in these factors over time, we assessed measurement invariance of the factor model. This was done by fitting the CFA model to the follow-up data and assessing model fit, to ensure that the model still provided a good fit to the data, and comparing factor loadings between time points to ensure that these were consistent across time points.

### Task

The task was taken from our previous study assessing relationships between loss, risk, and ambiguity aversion in the context of anxiety (Zbozinek et al., 2021). Gambling stimuli included two pairs of circles representing the left and right choices (see Figure 1). There were three versions of these choice pairs: a) Left (50%/50%), Right (100%), b) Left (100%), Right (50%/50%), and c) Left (100%), Right (100%). “50%/50%” indicates a 50% chance of receiving either of the two outcomes for that choice and was represented by a circle with a vertical line splitting it in half, and “100%” indicates a 100% chance of receiving that outcome and was represented by a circle. Gain amounts were color-coded as green, loss amounts as red, and 0 points as gray. Ambiguous outcome magnitudes were represented as “+?” or “-?” and color-coded as green or orange to represent gains or losses, respectively. For gain trials, points ranged from 10 to 90, while losses ranged from -90 to -10. Subjects 138 trials split across completed 7 blocks of the task. Between participants, block sequence was the same, but trial order was fully randomized within each block. Trial values were pre-determined in order to facilitate variability in gambling/not gambling decisions while maintaining consistency across subjects, and were identical to those in our previous work using this task (Zbozinek et al., 2021)

The task included 8 different conditions, each with a different combination of risk, gain/loss, and ambiguity. In Condition 1, subjects were given the choice of a mixed unambiguous gain/loss option or a guaranteed option. Condition 2 featured a mixed unambiguous gain/ambiguous loss and a guaranteed option. Condition 3 had a mixed ambiguous gain/unambiguous loss option and a guaranteed option. Conditions 4-6 were gain only, with both options never leading to a loss. Condition 4 had a mixed unambiguous gain/zero outcome and a guaranteed gain. Condition 5 featured a mixed ambiguous gain/unambiguous zero outcome and a guaranteed gain. Condition 6 gave subjects the choice of an unambiguous gain/zero option and an ambiguous gain. Conditions 7 and 8 removed the risk component of the decision. Condition 7 included an ambiguous guaranteed gain and an unambiguous guaranteed gain option. Condition 8 featured an ambiguous guaranteed loss and an unambiguous guaranteed loss option. All conditions are shown in Figure 1.

For the purposes of this study, we did not provide subjects with monetary incentives to perform well, as a pilot study implementing this economic decision-making task for small monetary rewards ($3-6) showed the effects of anxiety are minor or non-existent. This, coupled with other studies showing that more substantial monetary payments enhance risk aversion (Fehr-Duda et al., 2010; Holt & Laury, 2002; Markowitz, 1952) suggest that such payments were infeasible for a study of this size. Instead, we modified the task to make it more engaging, with the aim of encouraging investment in the outcomes in the absence of tangible rewards. To achieve this, we added a running count of points, which increased or decreased depending on the outcomes obtained in the task. Importantly, this meant subjects knew the outcome they had received on each trial, even if the option was presented as ambiguous. This meant that it was possible for subjects to estimate the value of the ambiguous outcome over time, an aspect which we account for in our modelling of behavior (described below). This count was reset at the beginning of each new block. Additionally, when a 50/50% gamble option was chosen, the outcome was visualized as a spinner that slowly settled on one option, making the task feel more like a game. This approach is similar to that used in prior studies using similar tasks, which demonstrated that effects such as risk aversion can be observed using “gamified” task variants without any monetary reward (Rutledge et al., 2014).

### Behavioral measures

We selected six behavioral measures to be used in further analysis, using the proportion of gambles as the variable of interest in each case (or proportion of ambiguous choices in conditions 7 and 8 where no gamble option was present). These measures were condition 4 as an index of risk aversion, the contrast of conditions 1 versus 4 as an index of loss aversion, condition 7 as an index of ambiguity aversion for sure gains, condition 8 as an index of ambiguity aversion for sure losses, conditions 1 versus 3 as an index of ambiguity aversion for risky gains, and conditions 1 versus 2 as an index of ambiguity aversion for risky losses. For consistency, all measures were coded such that higher scores indicate more aversion.

### Computational modelling of behavior

We fitted previously validated decision-making models incorporating risk aversion, loss aversion, and ambiguity aversion to our data. Five different decision-making models were defined. Decision model 1 used a traditional three-parameter prospect theory model, estimating risk aversion and loss aversion for each participant, but assuming no ambiguity aversion (Kahneman & Tversky, 2013; Sokol-Hessner et al., 2009, 2013; Tversky & Kahneman, 1992). Decision models 2 to 5 also included multiplicative ambiguous outcome magnitude parameters (i.e., “ambiguity parameters”): Model 2) one general ambiguity parameter (includes all six ambiguity Conditions), Model 3) separate ambiguous gain (Conditions 3, 5-7) and ambiguous loss (Conditions 2, 8) parameters, Model 4) loss context (Conditions 2, 3, 8) and no-loss context (Conditions 5-7) ambiguity parameters, and Model 5) ambiguous risky gain (Conditions 3, 5), risky loss (Condition 2), sure gain (Conditions 6-7), and sure loss (Condition 8) parameters.

Equations below are representative of the winning model, Model 5, which contains four ambiguity parameters estimated separately for risky gains (Conditions 3 & 5), sure gains (Conditions 6 & 7), risky losses (Condition 2), and sure losses (Condition 8). Ambiguity parameters were implemented as a multiplicative weight to the implied value of ambiguous amounts in each condition (the calculation of which is described in the following section). By assigning a weight to this implied value, we can determine whether participants overestimate or underestimate the value of ambiguous options and thus infer their preference or aversion to ambiguity.

For each trial, the subjective utilities (*u*) of the gamble and sure option were estimated according to the equations shown in Table 1.

Additional decision models were determined as follows. Decision model 1: traditional prospect theory model; α_rg_ = α_sg_ = α_rl_ = α_sl_ = 1 (no ambiguity preference or aversion). Decision model 2 featured a single ambiguity parameter; α_rg_ = α_sg_ = α_rl_ = α_sl_ = α. Decision model 3 included separate ambiguity parameters for gains and losses; α_rg_ = α_sg_ = α_g_ and α_rl_ = α_sl_ = α_l_.. Decision model 4 included separate ambiguity parameters for no-loss contexts (i.e. only values ≥$0 are present in the trial) and loss context (i.e. at least one loss is present in the trial).

This task differed from standard risky decision-making tasks in that the magnitudes of some options were ambiguous. In this case, the exact value of the option was not shown to participants, and they instead had to infer its value. In the variant of the task used here however, subjects were shown a running total of their points, which could be used to retrospectively infer the value of an ambiguous outcome on a given trial if it was chosen (i.e. the change in score represents the outcome of this trial). This meant that subjects could use this information to learn the value of the ambiguous options, and so we considered this possibility in our modelling. We approached this by incorporating a selection of learning models into our final model. Here, rather than providing the rational value as the value of the ambiguous option for the decision-making process, which is consistent across all trials, we instead provide it with the learned value from the learning model, which changes over the course of the task as subjects receive more information. We compared these learning models against a model that did not model learning of the ambiguous option, instead representing the value of the ambiguous option as the mean value of the unambiguous trials of the same type (for example, the implied value of an ambiguous loss was the mean value of all other loss values).

For each learning model, we tested three variants to capture initial biases in the perceived value of ambiguous options: The first (RW1, RW4, BMT1) set the initial value of the ambiguous option to 5 for gains, and -5 for losses. The second (RW2, RW5, BMT2) estimated this value as a free parameter, assuming the same absolute magnitude for ambiguous gains and losses, differing only in its sign. The final variant (RW3, RW6, BMT3) estimated the initial value of ambiguous options separately for gains and losses.

We focused on three families of learning models, one based on a standard Rescorla-Wagner model (RW 1, 2, 3), whereby the estimated value of the ambiguous gain and loss options (*V*) was updated on each trial (*t*) according to the prediction error (δ) weighted by a free learning rate parameter, α.

(1) $$\delta_{t}={\text{outcome}}_{\text{t}}-V_{\left\{t-1\right\}}$$

(2) $$V_{t+1}=V_{t}+\text{α}\cdot {\text{δ}}_{t}$$

The second was a derivative of the first Rescorla-Wagner model, but included separate estimated learning rates (α^−^,α^+^) for ambiguous losses and gains (RW 4, 5, 6).

(3) $${\text{V}}_{\left\{t+1\right\}}={\text{V}}_{t}+\begin{array}{c} {\text{α}}^{+}\cdot {\text{δ}}_{\text{t}}{\text{if}\;\text{δ}}_{t}>0\\ {\text{α}}^{-}\cdot {\text{δ}}_{\text{t}}{\text{if}\;\text{δ}}_{t}<0 \end{array}$$

The third model family was built on a Bayesian mean tracker model(Wu et al., 2018). This model estimates the mean and variance of the outcome distribution for ambiguous losses and gains (BMT 1, 2, 3). While the update step in the model is dependent on weighted prediction errors, as in the first two model families, the learning rate is adaptive and depends on the estimated variance of the distribution. As such, this model estimates uncertainty and adapts learning in response to it, independently for ambiguous losses and gains. The mean (*m*) and variance (*v*) of the option’s value are updated on each trial dependent on learning rate *G* as follows:

(4) $$m_{t}=m_{\left\{t-1\right\}}+\delta_{t}\cdot G_{t}$$

(5) $$v_{t}=\left(1-G_{t}\right)\cdot v_{\left\{t-1\right\}}$$

The learning rate *G* is updated on each trial as a function of the variance and an additional free parameter theta, which represents the error variance. Higher values of theta result in lower learning rates and lower uncertainty.

(6) $$G_{t}=\frac{v_{\left\{t-1\right\}}}{v_{\left\{t-1\right\}}+\theta_{\epsilon }^{2}}$$

Values were only updated on trials where outcomes were received (i.e. the value of ambiguous losses was only updated with an ambiguous loss was received, and vice versa for ambiguous gains). Subjective utility values were then passed through a softmax function to estimate the probability of choosing the gamble on each trial (coded as 1 or 0 for choosing the gamble or the alternative sure option, respectively), with the inverse temperature parameter γ:

(7) $$P(gamble\;)=\frac{1}{1+e^{-\text{γ}(u[gamble\;]-u[sure])}}$$

Gambles refer to the risky option (Conditions 1-6) or the ambiguous option (Conditions 7-8). Models were specified hierarchically (Valton et al., 2020) and fit in Python using variational inference implemented in PyMC3 (Salvatier et al., 2016, p. 3). We compared models using the widely available information criterion (WAIC), an index of model fit designed for Bayesian models, and calculated Efron’s *R*^2^ (Efron, 1978) to provide an indication of how well the model predicted subjects’ decisions We also provide a calibration plot (Figure S7) to demonstrate the correspondence between model-predicted choice probabilities and true choice probabilities. As these were Bayesian models, we approximated the full posterior distribution over parameter values, but we use the mean of this distribution as a point estimate of the parameter value for further analyses. For models with dynamic learning rates, we generated data from the model using 2000 samples from the approximate posterior distribution of our estimated parameters to produce a range of potential learning rate trajectories for each subject. For each sample, we calculated the mean learning rate across all trials, providing an indication of how subjects were learning over the course of the task. We then took the mean of these values across samples to represent a point estimate of the mean learning rate. For models with different learning rates for positive and negative outcomes, we subtracted the negative learning rate from the positive learning rate to provide a learning bias measure, which was used in further analyses. To ease interpretation, parameters were reversed as necessary (by subtracting each subject’s value from the maximum value across subjects) such that higher values represent aversion while lower values represent preference. For the computational modelling analyses, we excluded subjects who did not choose any ambiguous gain or loss options to enable us to fit models accounting for the learned value of these options accurately. Our analyses focused on parameters representing loss, risk, and ambiguity aversion, in addition to learning rate. Other parameters (for example softmax temperature parameters) were not included in further analyses.

To verify the accuracy with which we could infer the model used and its parameter values, we performed parameter and model recovery analyses (Wilson & Collins, 2019). This involved simulating data from the same number of subjects as completed the task with randomly chosen parameter values using each of the 50 models, and then fitting all models to each of these simulated datasets. To determine the likelihood of each model being the best fitting, we calculated model weights based on the WAIC using the stacking approach (Yao et al., 2018). For parameter recovery, we assessed the correlation between generated and recovered parameters in our winning model using Pearson correlation coefficients. We also verified the independence of parameters by calculating correlations between recovered parameter values. Results of these analyses are shown in Figure S4-6.

### Cross-sectional analysis

We assessed cross-sectional relationships between behavioral and questionnaire measures at baseline using latent path models, a form of structural equation model. This has the advantages of explicitly modelling latent factors and their relationship with observed variables, rather than simply taking estimated factor scores for each subject, and being able to comprehensively model covariance between variables of interest.

We incorporated the latent factors in the model using the same structure as was identified in the confirmatory factor analysis, with items loading on to the same factor as in the original factor analysis. Behavioral measures were modelled as latent variables, each loading on to their respective single observed variable with their loading fixed at 1. We included regression paths representing the latent factors as linear combinations of the behavioral variables, plus age and education level (as an index of socio-economic status) as covariates. Models were specified and estimated with Lavaan (Rosseel, 2012) in R 3.6.1 using maximum likelihood, and model fit was evaluated using the same measures as our confirmatory factor analysis. All reported parameter estimates are standardized. We report both uncorrected p-values and p-values corrected using the Benjamini-Hochberg FDR method, in line with best practices for multiple comparison correction in SEM (Cribbie, 2007) .

### Longitudinal analysis

We also used a structural equation modelling approach in analyzing the longitudinal data. Here, we used latent change score models (Kievit et al., 2018; McArdle, 2009) to assess whether behavioral measures at baseline predict change in factor scores over time. Again, this allowed us to model latent factors rather than using factor scores. Latent change score models also account for baseline levels of the measure of interest by modelling the time 2 variable as a combination of the time 1 score plus a latent variable representing the change between time points. This allowed us to examine predictive effects of behavioral measures through including regression terms between our behavioral variables and the latent change score, again including age and education level as covariates. By representing the change between timepoints as a latent variable and predicting time 2 scores as a combination of time 1 scores and this latent change variable, this directly assesses whether behavioral variables predict change in the factors of interest, accounting for baseline levels of these factors. We fitted separate models for each factor. Models were fit using Lavaan in R 3.6.1. As with the cross sectional models, we report uncorrected and correct p-values (corrected across all the latent change score models used).

## Results

### Sample characteristics

Of the 479 subjects recruited, we excluded 16 subjects who did not choose any ambiguous options and 11 who were deemed multivariate outliers based on questionnaire data, leaving a total of 452 subjects. This group had a mean age of 33.56 (SD=11.78), although 7 subjects did not report age. Of these, 171 reported their sex as male, 267 as female, and 14 did not report sex. For exploratory factor analysis, we used data from an additional 1134 subjects who did not complete the task. 33 of these were excluded, and the resulting sample had a mean age of 32.85 (SD=12.58, 44 not reported), with 449 males, 558 females, and 72 not reporting sex. Of the original 479 subjects, 260 completed questionnaire measures at Time 2, with a mean age of 34.43 (SD=12.27, 4 not reported). Of these, 104 were male, 148 female, and 8 did not report sex.

We ensured that there were no systematic differences at baseline between the subjects who completed the follow-up and those who did not. We tested this using a logistic regression predicting follow-up status (completed vs dropped out) from factor scores at T1, behavioral variables, and age. None of these variables signficiantly predicted whether subjects completed the second time point (Table S4), indicating that subjects who dropped out were not qualitatively different from those who did not.

### Measures of psychological and behavioral responses to the pandemic

Taken as a whole, the questionnaire battery used demonstrated high internal consistency, with an alpha value of 0.91 and an omega of 0.95. Exploratory factor analysis was conducted in an independent sample of 1101 subjects who responded to questionnaires at Time 1 but were not asked to complete the gambling task, allowing us to subsequently confirm the factor structure in those who did complete the task. Barlett’s test of sphericity in this independent sample indicated that the data were appropriate for factor analysis (*X*^2^ (276) =14161.94, *p* < .001). The scree plot and fit statistics indicated that a five-factor solution was optimal, and the root mean squared error approximation (RMSEA) was 0.07, indicating good fit (Rigdon, 1996). These factors broadly related to general anxiety, virus-related anxiety, engagement in protective behaviors, perceived infection likelihood, and perceived infection severity. Factor loadings are shown in Figure 2C and Table S2.

Confirmatory factor analysis was run to evaluate the fit of this five-factor model in the sample who completed the task at Time 1. This model provided good fit to the data (*X*^2^ (125) = 370.27, p<.001; CFI=.94; RMSEA=.07, standardized root mean square residual (SRMR)=.06). Factor loadings for this model are provided in Table S3. We also fit this model to the Time 2 data to assess measurement invariance, fixing factor loadings and intercepts to values estimated at Time 1. The model retained a good fit at Time 2 relative to Time 1 (ΔCFI=.009; ΔRMSEA=.003), and this was similar when fixing residual estimates to those identified at Time (ΔCFI=.01; ΔRMSEA=.002), providing evidence of measurement invariance across time points.

### Aversion to loss, risk, and ambiguity

We first examined the extent to which subjects as a whole were averse to risk, loss and ambiguity through assessing proportions of gambles and ambiguous choices made in the gambling task (Figure 2B). Overall, subjects demonstrated a slight risk preference, as assessed by the number of gambles chosen, likely due to the low stakes involved (*t* (478) = -6.77, *p* < .001). However, they demonstrated a tendency to be loss averse (*t* (478) = 16.69, *p* < .001), as indicated by a comparison of conditions 1 (gamble with loss outcome) and 4 (gamble without loss outcome), and ambiguity averse (*t* (478) = 14.40, *p* < .001), as demonstrated by a comparison of conditions 4 (gamble with known sure outcome) and 6 (gamble with ambiguous sure outcome).

### Computational modelling of learning and decision making

We used computational modelling to provide a more fine-grained picture of how decision-making processes might relate to risk perception and anxiety in response to a real-world stressor. This allowed us to explore decision making and learning processes across our experimental conditions, making full use of the rich behavioral data provided by the task. Because subjects saw the outcome of their choice on each trial, this raised the possibility that subjects could learn the approximate value of the ambiguous options over the course of the task. For this reason, we included models that accounted for this learning process.

**Figure 3:**
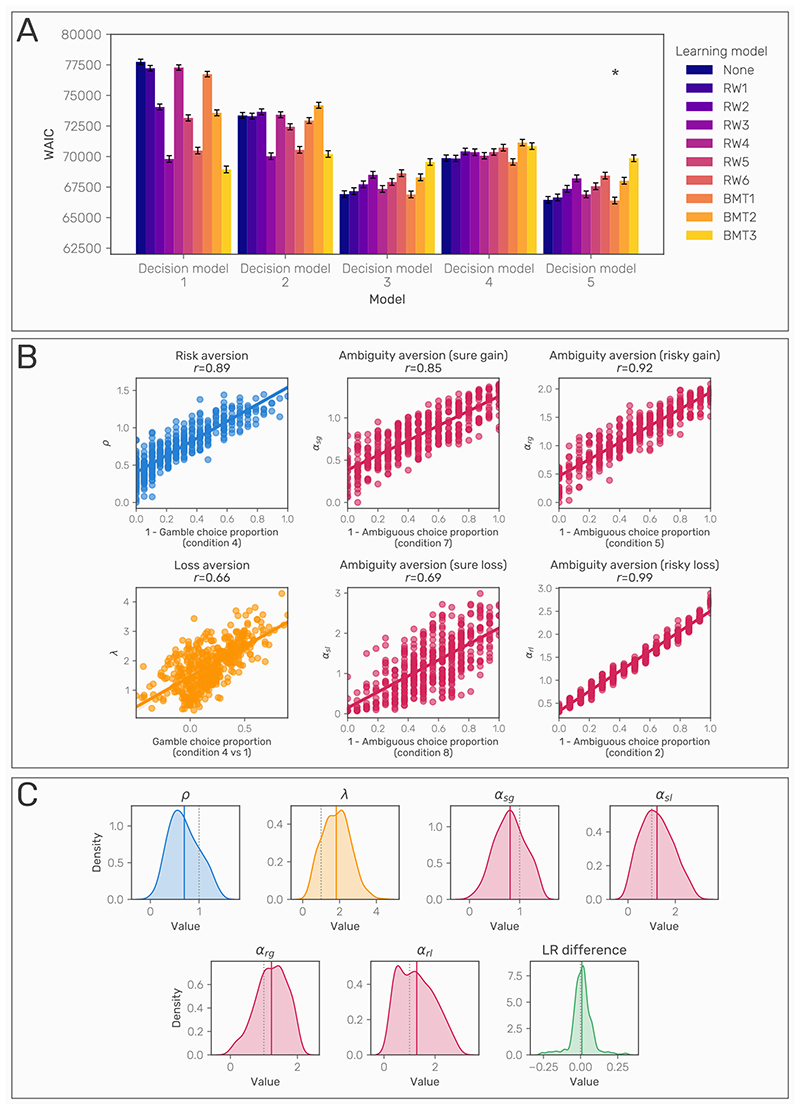
A) Model comparison results, showing WAIC scores for each candidate combination of decision and learning models (lower scores represent better fit). Models are grouped on the X axis according to the decision model used, while bars within these groups represent the learning model used. RW: Rescorla Wagner, BMT: Bayesian Mean Tracker. B) Relationships between model-agnostic measures of decision making processes and parameters from the winning model. C) Distributions of estimated parameter values. The dotted gray line indicates the value at which no bias in either decision making or learning is present, while the solid line indicates the mean value across subjects. For parameters in the decision model (*ρ*, *λ*, *α*) higher values indicate aversion, while for the learning rate difference higher values indicate faster learning from gains relative to losses.

Our modelling approach involved testing five decision models and nine learning models (plus decision making models in the absence of any learning), whereby the learning model served to estimate values of the ambiguous options while the decision-making model translated observed and learned values into choices. The best fitting combination of models comprised a Bayesian mean tracker learning model in conjunction with a decision making model with separate ambiguity aversion parameters for each four combinations of outcome amount (win/loss) and risk level (risky/sure) (Figure 3A), with a pseudo-*R*^2^ value of 0.15. Parameter estimates from this model (Figure 3C) were strongly correlated with associated behavioral measures (all *r*s > .65), providing confidence in their validity (Figure 3B), and parameter recovery tests indicated that we were able to recover parameter values accurately (Figure S4).

### Cross-sectional associations between pandemic responses and decision making

Next, we investigated how the decisions subjects made in the task related to their perceived threat from the pandemic, alongside pandemic-related anxiety and general anxiety in the past week. These analyses allowed us to assess how decision-making styles related to immediate reactions to stress. We excluded 7 subjects from these analyses as they did not report age, which was included as a covariate in all models. Thus, the final sample consisted of 445 subjects.

**Figure 4:**
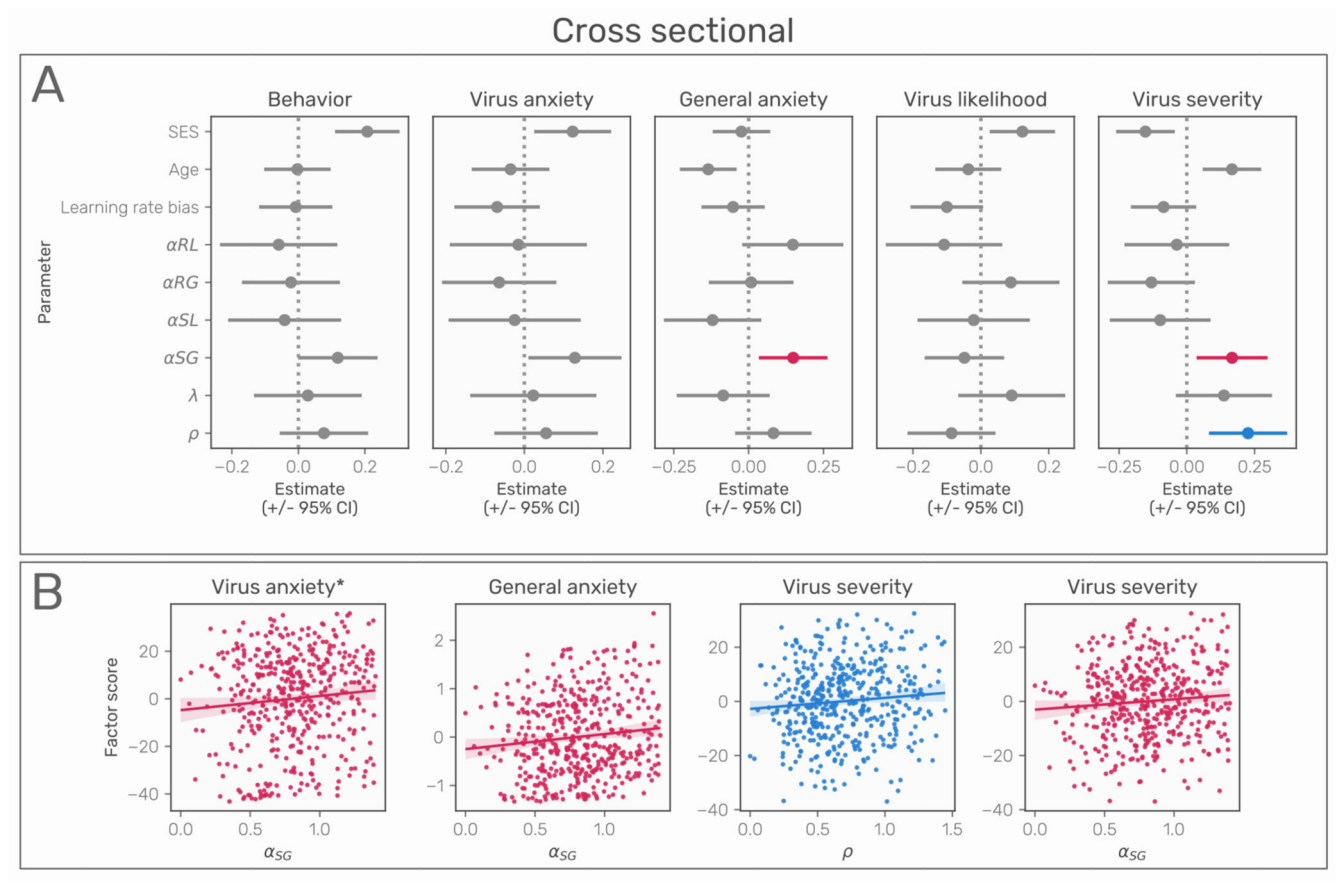
Predictors of psychological and behavioral factor scores. A) Effects of (model ( parameters on each of the five factors identified through factor analysis. Estimates represent standardized parameter estimates from a latent path model using variables measured at Time 1. Significant effects, corrected for multiple comparisons, are highlighted. B) Scatter plots showing significant relationships between decision-making variables and self-report measures. Regression lines are plotted for illustration and were not used for statistical inference, as they do not account for other variables included in the full model. The effect on virus anxiety is shown for illustration but does not survive correction for multiple comparisons.

We assessed how decision-making relates to the identified latent factors underlying psychological and behavioral responses to the pandemic using (parameters from the computational model (referred to as model-based). Results are shown in Figure 4). We observed the strongest effects with ambiguity aversion for sure gains. Greater model-derived ambiguity for sure gains was associated with greater general anxiety (*β*=0.15, 95% CI=[0.05, 0.26], *p* = .01, *p_corr_* = .02), virus-anxiety, although this did not survive correction for multiple comparisons, (*β*=0.13, 95% CI=[0.001, 0.25], *p* = .033, *p_corr_* = .05), and perceived virus infection severity (*β*=0.17, 95% CI=[0.04, 0.30], *p* = .01, *p_corr_* = .02). ( (We also found a significant positive relationship between risk aversion and perceived infection severity, where subjects who were more risk averse perceived potential infection to be more severe (*β*=0.23, 95% CI=[0.08, 0.37], *p* = .002, *p_corr_* = .004).

### Prediction of longitudinal changes in pandemic responses

Next, we examined longitudinal changes in our psychological and behavioral factors over time. To assess the extent to which scores on these measures changed over time, we extracted latent change scores for each subject, testing these for significance using one-sample t-tests against zero. At the group level, we observed moderate increases in virus-related anxiety (*t*(259)=4.91, *p*<.001, *d*=0.31) and perceived infection severity (*t*(259)=7.51, *p*<.001, *d*=0.47). Engagement in protective behaviors, however, showed a much larger increase (*t*(259)=20.03, *p*<.001, *d*=1.24). No significant change over time was observed in general anxiety (*t*(259)=0.95, *p*=.34, *d*=0.11) or perceived infection likelihood (*t*(259)=-.68, *p*=.50, *d*=0.09). Changes in these measures, along with distributions of latent change scores, are shown in Figure 2D.

**Figure 5:**
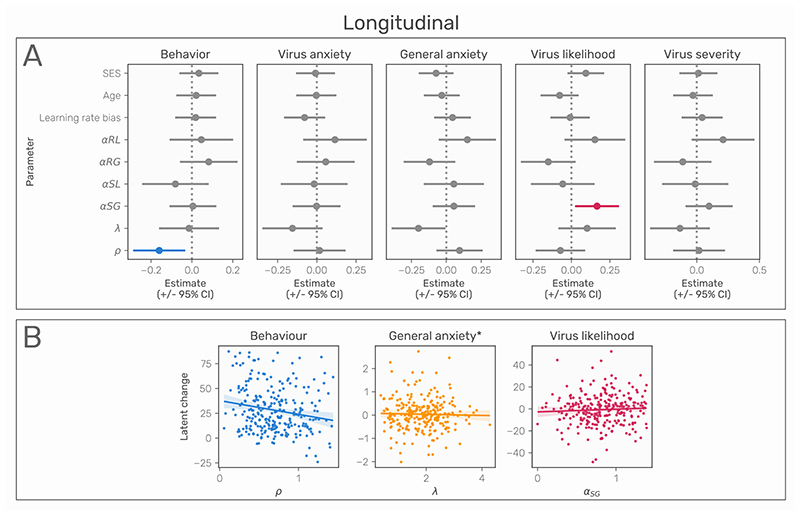
Predictors of longitudinal changes in psychological and behavioral factor scores. A) Effects of model parameters on change in the variables identified through factor analysis. Estimates represent standardized parameter estimates from a latent change score model, predicting change scores from behavioral variables measured at Time 1. Significant effects, corrected for multiple comparisons, are highlighted. B) Scatter plots showing significant relationships between decision-making variables and changes in self-report measures, with latent change scores derived from the model plotted on the Y axis. Regression lines are plotted for illustration and were not used for statistical inference, as they do not account for other variables included in the full model. The effect on general anxiety is shown for illustration but does not survive correction for multiple comparisons.

We then assessed effects of behavioral variables on these latent change scores (Figure 5). This revealed multiple significant effects. First, loss aversion was negatively associated with changes in general anxiety, although this did not survive correction for multiple comparisons (*β*=-0.20, 95% CI=-[0.39, -0.007], *p* = .04, *p*_corr_ = .06), providing some evidence that individuals who were less loss averse at Time 1 became more anxious over time. We also found that risk aversion was negatively associated with changes in protective behaviors (*β*=-0.16, 95% CI=[-0.29, -0.03], *p* = .01, *p*_corr_ = .02), indicating that individuals who were more risk-seeking at Time 1 increased their engagement in protective behavior to a greater extent. The only positive association was found between ambiguity aversion for sure gains and perceived likelihood of infection, where individuals who were more ambiguity-averse at Time 1 perceived infection to be more likely at Time 2 compared to Time 1 (*β*=0.17, 95% CI=[0.02, 0.24], *p* = .02, *p*_corr_ = .03).

## Discussion

We investigated relationships between decision making under uncertainty and emotional responses to a real-world stressor: the COVID-19 pandemic. We show that biases in low-level decision-making processes manifesting in situations involving uncertainty predict psychological and behavioral reactions to stress both at its onset and longitudinally, as the stressor persists. These results represent a rare window into the processes that influence emotional reactions to a salient real-world stressor in the short and medium term.

Cross-sectionally, at the onset of the pandemic, we found that ambiguity aversion, particularly in situations with guaranteed gains, was associated with heightened anxiety related to the pandemic and greater perceived infection severity. This suggests that individuals who are less comfortable with ambiguous situations may have more extreme perceptions of stressful events and become more anxious about them, echoing results from work on the moderating role of intolerance uncertainty on reactions to stress (Chen & Hong, 2010; Oglesby et al., 2016). It is intriguing that this effect was specific to guaranteed gains, as this suggests that it is perceptions of positive outcomes that drives these reactions, when responses to negative outcomes are frequently implicated in anxiety (Aylward et al., 2019; Browning et al., 2015; Wise et al., 2019; Wise & Dolan, 2019). We found similar results with risk aversion, whereby subjects who were more risk averse also perceived infection severity to be greater, pointing to a role of risk aversion in risk perception. Notably, in contrast with prior work (Charpentier et al., 2017), we found no effects relating risk aversion to general anxiety. This may be due to our focus on past-week anxiety, while previous work in this area has typically used samples of clinically anxious individuals and measured anxiety over longer periods.

Our longitudinal data, focusing on change in responses over time, depicted a more complex pattern. We again found an effect of ambiguity aversion for sure gains, whereby more ambiguity averse subjects’ perception of infection likelihood increased over time. However, we also found negative effects of risk and loss aversion on change in protective behaviors and general anxiety, respectively. These results are somewhat counter-intuitive, indicating that more risk-seeking subjects increased their engagement in protective, avoidant behaviors more over time, while subjects who were initially less loss-averse became generally more anxious over time. Importantly, these analyses controlled for baseline levels of these variables, focusing instead on the change over time that is not explained by baseline measures, meaning that these results are not confounded by higher baseline scores. This is further bolstered by a lack of association at baseline between risk aversion and protective behaviors, and loss aversion and general anxiety, suggesting this effect indeed reflects a prospective effect.

The use of both cross-sectional and longitudinal analyses allows us to answer two related but different questions. The first focuses on immediate levels of risk perception, anxiety, and behavior. The latter focuses on *changes* in these factors. Based on the timing of the study, we can interpret the cross-sectional results showing associations between decision-making processes and a form of “default” level of risk perception that individuals adopt in the absence of concrete experience or information. At the baseline timepoint, the COVID-19 pandemic was in its infancy in the United States. As a result, most individuals likely had not acquired a great deal of information about it and were therefore unable to form a robust estimate of risk. In this context, we might expect more risk averse individuals to perceive greater threat from the pandemic, while more ambiguity averse individuals react negatively to the uncertainty about the situation with greater anxiety, as observed. Over time however, this ambiguity aversion may prompt individuals to seek information about the pandemic, increasing their perception of risk. Additionally, if more risk averse individuals perceive likelihood of infection to be higher in the early stage of the pandemic, they may engage in protective behavior sooner (in the time between the two time points studied here). This may result in fatigue or complacency over time, leading to relatively lower engagement at a later timepoint.

These possible interpretations will require further testing. There may also be more complex effects involved, for example it is possible that individuals with higher risk preference may increase engagement in protective behavior over time because, due to genetic or social factors for example, they share an environment with others who are more risk seeking and therefore need to protect themselves from likely sources of infection. One broad important implication of these results is that care should be taken when extrapolating from associations identified in cross sectional work to longitudinal settings. Alternatively, this may reflect more less risk averse individuals having greater psychological flexibility, allowing them to adapt their behavior over time.

Our results indicate that responses to a real-world stressor are associated with individual differences in decision making processes and provide suggestive evidence that these processes could play a role in mediating the relationship between stressful events and anxiety. It should be noted that the effects seen here are relatively small. This should not be surprising however, as it is likely that decision making processes do not explain a large proportion of the variance in responses to a stressful life event on the scale of a pandemic, which will be largely dominated by factors such as health, employment conditions, and social network. Nevertheless, these effects provide robust evidence for a small but meaningful influence of these processes.

Furthermore, this is the first study to our knowledge to experimentally control and manipulate ambiguous outcome magnitude for both gains and losses. Previous studies have generally confounded ambiguous outcome magnitude and likelihood or focused on ambiguous likelihood. This study therefore provides insight into clinical notions of “catastrophizing” and suggests that ambiguous outcome magnitude is indeed associated with anxiety, though in the gain rather than loss domain. Future research can test the durability of this effect. Moreover, while our computational model also included a learning component, we did not see any effect of learning rates on our self-report measures. This may be somewhat surprising, as it might be expected that risk perception would be influenced by learning styles. However, this task was not specifically designed to measure value learning, so it is possible that this null result simply reflects the low sensitivity of the task used.

However, our study has several important strengths. First, we studied reactions to a stressful life event on a scale rarely experienced by most individuals, and this therefore represents a rare view of responses to a major stressor. This allowed us to test how processes measured using behavioral tasks predict response to real-world stress, as opposed to acute stress often administered in the lab. Second, we evaluated changes over time, allowing us to determine how decision-making processes relate to the evolution of anxiety responses as the stressor develops. Third, our use of online testing enabled us to recruit a large sample across the United States, increasing generalizability of our findings. Finally, our data collection began on March 14, 2020, which was early in the pandemic in the US. This was three days after the World Health Organization officially announced that COVID-19 was a pandemic, and there were 2,174 cumulative cases and 47 cumulative deaths in the US. Collecting data early in the pandemic is an advantage as it provides somewhat of a baseline prior to the drastic changes in health, lifestyle, economics, and mental health that occurred due to the pandemic. Finally, there were no validated measures of the investigated constructs available when we began the study, the analyses presented here indicate that these measures have good psychometric properties, including high internal consistency of the identified latent factors, which is bolstered by their consistency across two independent samples and across 8 weeks.

Despite the strengths of our study, some limitations should be considered. First, while we controlled for socioeconomic status, there are likely to be other factors related to unemployment and health effects, for example, that influenced the extent to which individuals were affected by the COVID-19 pandemic. These effects are likely to be magnified further for members of marginalized communities (Czeisler et al., 2020; Fortuna et al., 2020). Second, our sample was recruited online and therefore is unlikely to be fully representative, although online studies are typically more representative that those conducted on college students (Berinsky et al., 2012). Third, our decision-making task was based on scoring ‘points’, rather than meaningfully large dollar amounts, and did not include monetary incentives which may have encouraged risk preference. Fourth, our decision-making task showed a cumulative performance score, which may have affected decision-making processes based on recent or distal outcomes. However, our large sample should mean that any such effects are diluted if they are not systematically associated with our outcome variables of interest.

Our results show that low-level uncertainty aversion is tightly linked to perceptions of risk and anxiety in response to major real-world stress. These findings identify a key role for decision making under uncertainty in emotional reactions to real-world stress and highlight the benefits of assessing stress responses through a more computational lens.

## Supplementary Material

Supplementary material

## Figures and Tables

**Table 1:** Computational Modeling Calculations

Condition	u (gamble’) =	u(sure) =
1: Mixed gain/loss, unambiguous	0.5 * *gain^**p**^* + 0.5 * ***λ*** * (− *loss*)^**ρ**^	0
2: Mixed gain/loss, ambiguous risky loss	0.5 * *gain^**ρ**^* + 0.5 * ***λ*** * ***a_rl_*** * (*V_ASL_*)*^**ρ**^*	0
3: Mixed gain/loss, ambiguous risky gain	0.5 * ***α_rg_*** * *V_ARG_^**ρ**^* + 0.5 * *****λ***** * (− *loss*)^***ρ***^	0
4: No-loss, unambiguous	0.5 * *gain^**ρ**^*	*sureG^**ρ**^*
5: No-loss, ambiguous risky gain	^0^.^5^ * ***α_rg_*** * *V_ARG_^**ρ**^*	*sureG^**ρ**^*
6: No-loss, ambiguous sure gain	0.5 * *gain^**ρ**^*	***α_sg_*** * ***V**_asg_^**ρ**^*
7: No risk, ambiguous sure gain	***α_sg_*** * ***V**_ASG_^**ρ**^*	*sureG^**ρ**^*
8: No risk, ambiguous sure loss	***λ*** * ***α**_**sl**_* *V*_ASL_^***ρ***^	**λ** * sureL^**ρ**^

*λ (“lambda”) represents loss aversion (where λ > 1 indicates overweighting of losses relative to gains, and λ < 1 indicates underweighting losses relative to gains). ρ (“rho”) represents the curvature of the utility function, which reflects varying sensitivity to changes in values as value increases. If ρ < 1, the utility function is concave for gains and convex for losses, indicating risk aversion (i.e., less utility for a gamble than a sure option with the same expected value); ρ > 1 indicates risk-seeking. αrg, αsg, αrl, and αsl (“alpha risky gain,” “alpha sure gain,” “alpha risky loss,” and “alpha sure loss,” respectively) represent the ambiguity parameters for risky gains, sure gains, risky losses and sure losses, respectively. In the case of gains (αrg and αsg), values < 1 mean that ambiguous gain values are underestimated compared to the rational gain, indicating ambiguity aversion, while values > 1 indicate ambiguity preference. In the case of losses (α_rg_ and α_sg_), values > 1 mean that ambiguous loss values are overestimated compared to the rational loss, indicating ambiguity aversion, while values < 1 indicate ambiguity preference. In other words, for parameters with “Aversion” in their name (e.g., Ambiguous Risky Loss Aversion), values > 1 indicate ambiguity aversion, whereas values < 1 indicate preference. Conversely, for parameters with “Preference” in their name (e.g., Ambiguous Risky Gain Preference), values > 1 indicate ambiguity preference, whereas values < 1 indicate ambiguity aversion. For ease of interpretation, the values of these parameters were rescaled such that higher values indicate aversion in further analyses. V values represent implied values of ambiguous options, which are either learned or calculated from observed unambiguous options.*

**Table 2:** Decision models

Decision model	Description
1	α_rg_ = α_sg_ = αrl = α_sl_ = 1 (no ambiguity preference or aversion)
2	α_rg_ = α_sg_ = α_rl_ = α_sl_ = α (single ambiguity parameter)
3	α_rg_ = α_sg_ = α_g_, α_rl_ = α_sl_ = α_l_ (separate ambiguity parameters for loss and gain)
4	α_l_ if loss present in trial, α_g_ if no loss present in trial
5	α_rg_, α_sg_, α_rl_, α_sl_ (separate ambiguity parameters for loss and gain, risky and safe)

**Table 3:** Learning models

Learning model	Learning algorithm	Adaptive learning rate	Asymmetry	Starting value
1. None	No learning about ambiguous outcome value	N/A	N/A	N/A
2. RW1	Rescorla-Wagner	X	X	Fixed: gains = 5, losses = -5
3. RW2	Rescorla-Wagner	X	X	Estimated: same value for gains & losses
4. RW3	Rescorla-Wagner	X	X	Estimated: separate value for gains & losses
5. RW4	Rescorla-Wagner	X	✓	Gains = 5, losses = -5
6. RW5	Rescorla-Wagner	X	✓	Estimated: same value for gains & losses
7. RW6	Rescorla-Wagner	X	✓	Estimated: separate value for gains & losses
8. BMT1	Bayesian mean tracker	✓	✓	Fixed: gains = 5, losses = -5
9. BMT2	Bayesian mean tracker	✓	✓	Estimated: same value for gains & losses
10. BMT3	Bayesian mean tracker	✓	✓	Estimated: separate value for gains & losses

## Data Availability

Data is available at https://osf.io/jgpex/ (Wise, 2021) and code is available at https://github.com/tobywise/uncertainty-pandemic-threat-perception. This work was not preregistered.
