# Supplementary material for: Computationally-defined markers of uncertainty aversion predict emotional responses during a global pandemic
Source: Emotion. Author manuscript; Available in PMC 2023 Apr 21. (PMC9942526; doi:10.1037/emo0001088)
Supplement: Supplementary material [file EMS140893-supplement-Supplementary_material.pdf]

Computationally-defined markers of uncertainty aversion predict emotional responses during a global pandemic

Toby Wise[1,2*], Tomislav D Zbozinek[1*], Caroline J. Charpentier[1,5], Giorgia Michelini[3,4], Cindy C Hagan[1] & Dean Mobbs[1,6]

[1]Division of Humanities and Social Sciences, California Institute of Technology, Pasadena, CA
[2]Department of Neuroimaging, Institute of Psychiatry, Psychology and Neuroscience, King's College London, London, UK
[3]Department of Psychology, Queen Mary University of London, London, UK
[4]Semel Institute for Neuroscience and Human Behavior, University of California Los Angeles, Los Angeles, CA
[5]Institute of Cognitive Neuroscience, University College London, London, UK
[6]Computational Neural Systems Program, California Institute of Technology, Pasadena, CA
*These authors contributed equally

Supplementary materials

Table S1. Items used to measure psychological and behavioral responses.

Question ID	Item
1	How likely do you think you are to catch the virus?
2	How badly do you think your health will be affected if you do catch the virus?
3	How badly do you think you will be affected economically if you specifically catch the virus (for example through loss of work)?
4	How badly do you think you will be affected by the global effects of the virus (for example economic recession, reduced healthcare capacity)?
5	How likely do you think it is that a loved one will become infected?
6	How likely do you think the average person in your neighborhood is to become infected?
8	How likely do you think the average person in the USA is to become infected?
9	If you do contract the virus, how likely do you think it is that you will pass it on to someone else?
10	If you do contract the virus and pass it on to someone else, how badly do you think they would be affected?
12	How scared would you be if a person coughed near you?
14	How often do you feel anxious about the virus?
26	The virus has caused me to avoid in-person social interactions (e.g., friends, family, co-workers, strangers).
29	I am washing my hands more than I would normally
30	I am staying home more than I normally would
31	I am travelling less than I would normally
32	I am aware of how much I touch my face more than I normally am
33	If I cough, I get worried
34	If I feel like I have a fever, I get worried.
35	If I feel short of breath, I get worried.
48	In the past week, how often have you felt anxious?
49	In the past week, when you have felt anxious, how intense or severe was your anxiety?
50	In the past week, how often did you avoid situations, places, objects, or activities because of anxiety or fear?
51	In the past week, how much did your anxiety interfere with your ability to do the things you needed to do at work, at school, or at home?
52	In the past week, how much has anxiety interfered with your social life and relationships?

Table S2. Factor loadings from exploratory factor analysis. Items retained for confirmatory factor analysis based on loadings of > .5 are highlighted in bold.

Question ID	General anxiety	Virus likelihood	Behavior	Virus anxiety	Virus severity
1	0.06	**0.74**	0.02	0.09	-0.08
2	-0.01	-0.01	-0.08	0.08	**0.62**
3	0.05	-0.04	0.03	-0.06	**0.51**
4	0.06	0.16	0.08	-0.02	0.38
5	-0.01	**0.82**	-0.01	0.00	-0.04
6	-0.01	**0.87**	0.04	-0.03	0.02
8	-0.02	**0.70**	-0.02	-0.07	0.15
9	0.02	0.29	-0.08	0.13	0.04
10	0.02	0.16	-0.05	0.04	**0.54**
12	0.01	0.05	0.16	0.32	0.44
14	0.23	0.12	0.18	0.31	0.27
26	0.11	0.07	**0.57**	0.09	0.14
29	-0.02	0.06	0.44	0.30	-0.09
30	-0.00	0.03	**0.87**	-0.01	-0.02
31	-0.01	-0.01	**0.81**	0.02	-0.03
32	0.00	0.12	0.28	0.30	-0.04
33	0.10	-0.02	0.07	**0.62**	0.17
34	-0.02	0.01	0.03	**0.85**	-0.01
35	0.02	0.01	0.00	**0.79**	-0.00
48	**0.86**	0.07	-0.08	0.11	-0.06
49	**0.90**	0.03	-0.07	0.03	-0.08
50	**0.59**	0.02	0.25	-0.03	0.11
51	**0.83**	-0.05	0.08	-0.07	0.07
52	**0.76**	-0.07	0.04	-0.06	0.08

Table S3. Factor loadings from confirmatory factor analysis. The first loading for each factor is fixed at 1.

Latent Factor	Question ID	B	SE	Z	Beta
General anxiety	48	1.00	0.00	N/A	0.83
General anxiety	49	0.88	0.04	20.64	0.84
General anxiety	50	0.86	0.05	16.75	0.72
General anxiety	51	0.90	0.05	19.99	0.82
General anxiety	52	0.81	0.05	16.34	0.71
Virus likelihood	1	1.00	0.00	N/A	0.77
Virus likelihood	5	1.08	0.06	18.31	0.83
Virus likelihood	6	1.15	0.06	19.30	0.89
Virus likelihood	8	0.85	0.06	14.28	0.67
Behavior	26	1.00	0.00	N/A	0.72
Behavior	30	1.26	0.09	14.80	0.82
Behavior	31	1.21	0.08	14.52	0.79
Virus anxiety	33	1.00	0.00	N/A	0.69
Virus anxiety	34	1.27	0.08	15.59	0.88
Virus anxiety	35	1.21	0.08	15.24	0.82
Virus severity	2	1.00	0.00	N/A	0.61
Virus severity	3	0.96	0.13	7.36	0.46
Virus severity	10	1.10	0.13	8.34	0.78

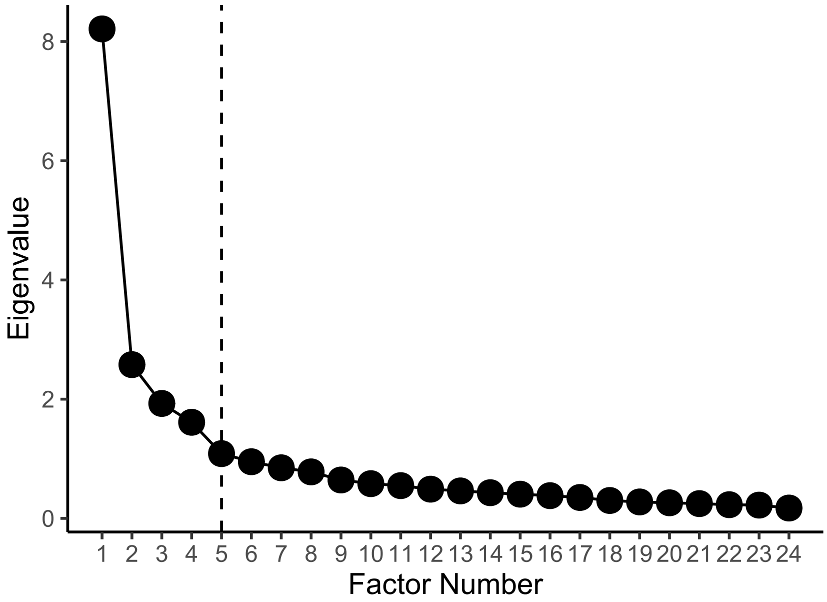

Figure S1. Scree plot showing eigenvalues from exploratory factor analysis.

Table S4. Results of logistic regression predicting follow up status from variables at baseline.

	Estimate	Standard Error	Z	p
(Intercept)	-0.224	0.312	-0.716	0.4738
General anxiety	0.140	0.161	0.872	0.3832
Virus likelihood	-0.003	0.006	-0.421	0.6738
Behavior	0.001	0.006	0.211	0.8329
Virus anxiety	0.004	0.007	0.554	0.5799
Virus severity	-0.004	0.008	-0.491	0.6237
Risk aversion	-0.523	0.447	-1.171	0.2417
Loss aversion	-0.034	0.220	-0.155	0.8772
Ambiguity aversion (SG)	-0.484	0.416	-1.165	0.2440
Ambiguity aversion (SL)	0.447	0.255	1.752	0.0798
Ambiguity aversion (RG)	0.572	0.324	1.769	0.0768
Ambiguity aversion (RL)	-0.272	0.258	-1.055	0.2913
Learning rate difference	0.024	1.545	0.015	0.9877
Age	0.017	0.009	1.874	0.0610

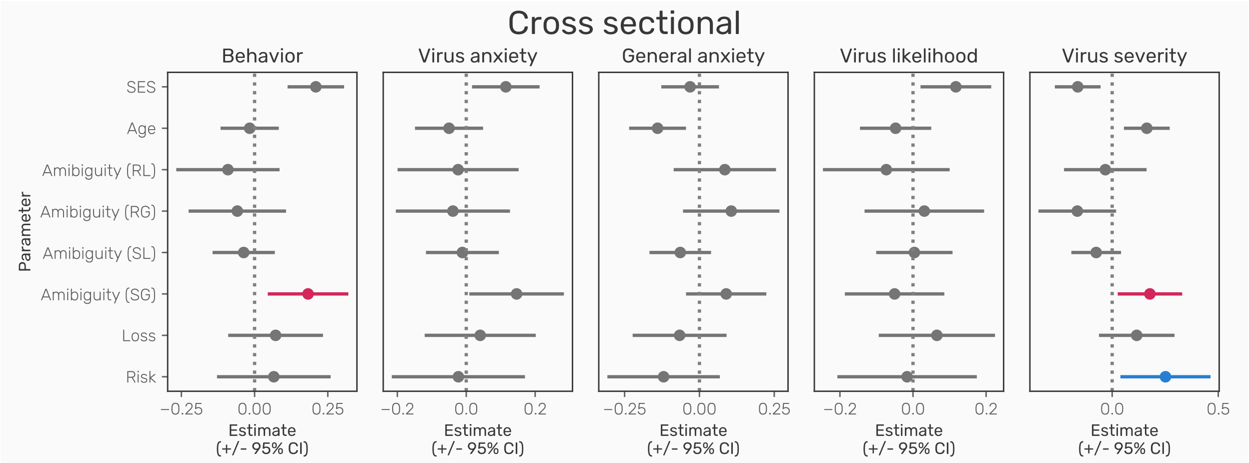

Figure S2. Results of cross sectional analysis using model-agnostic decision-making measures. Parameters on the Y axis correspond to model-agnostic measures of loss, risk, and ambiguity aversion. Ambiguity aversion is separated into sure gains (SG), sure losses (SL), risky gains (RG) and risky losses (RL).

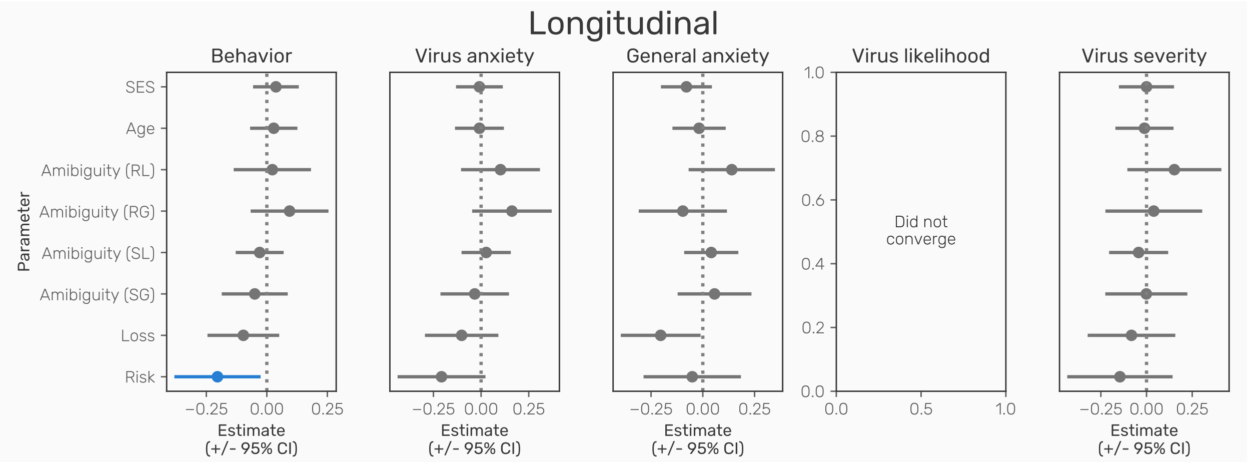

Figure S3. Results of longitudinal analysis using model-agnostic decision-making measures. Parameters on the Y axis correspond to model-agnostic measures of loss, risk, and ambiguity aversion. Ambiguity aversion is separated into sure gains (SG), sure losses (SL), risky gains (RG) and risky losses (RL).

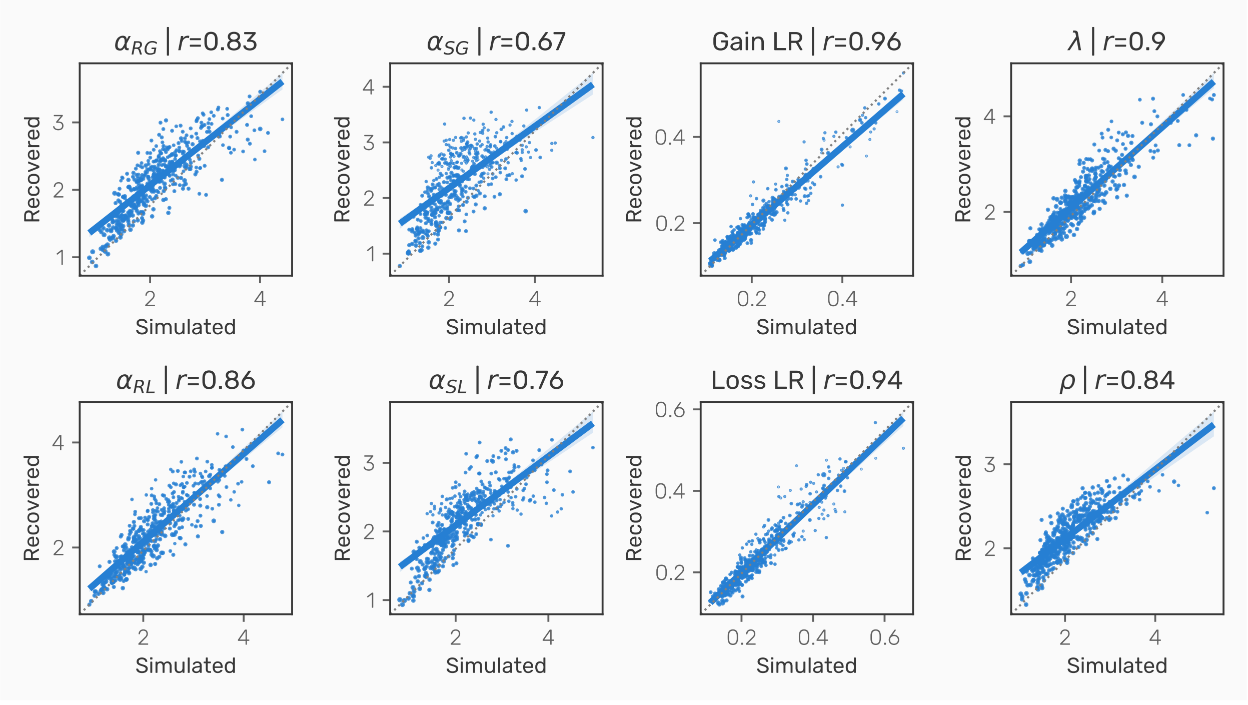

Figure S4. Results of parameter recovery, showing correlations between parameter values used to simulate data on the x-axis and recovered parameter values on the y axis.

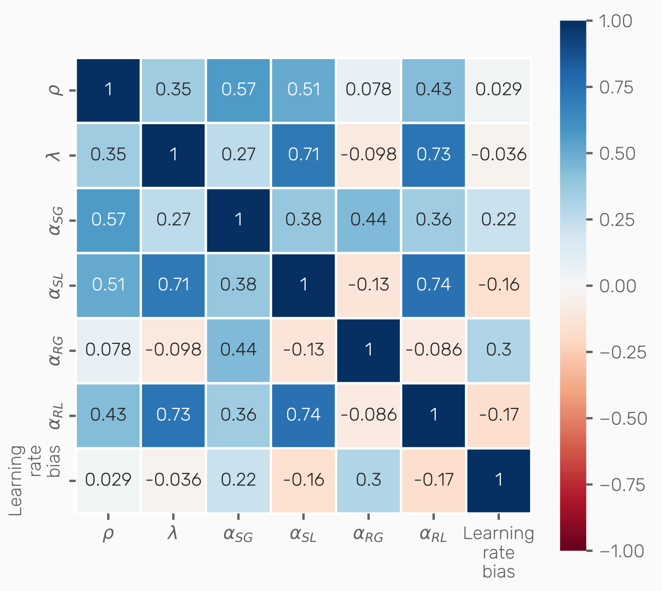

Figure S5. Correlations between parameter values.

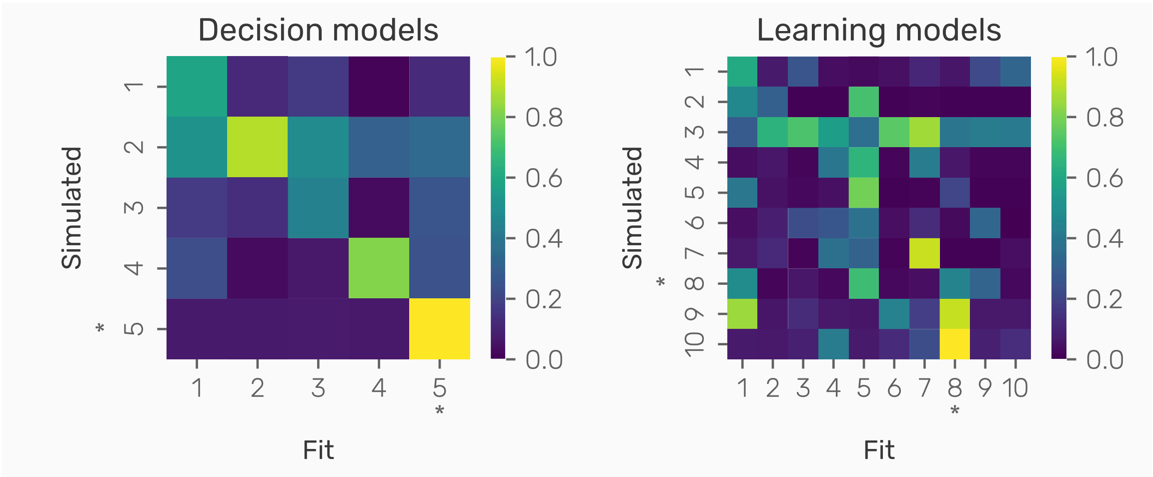

Figure S6. Results of model recovery analysis, showing the probability of each model being the best fitting model when fit to simulated data from each of the models. A) Results for decision models, collapsed across learning models. B) Results for learning models, collapsed across decision models.

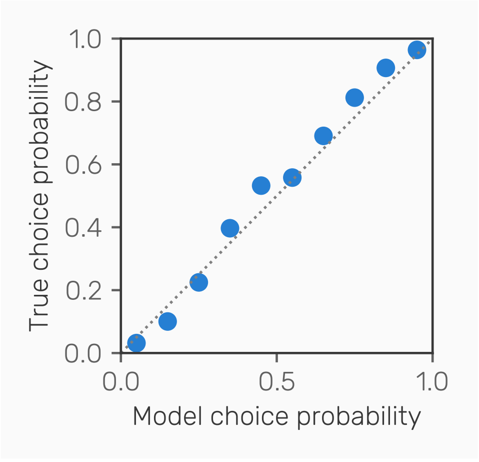

Figure S7. Calibration plot demonstrating correspondence between model predicted gamble probability (binned using intervals of 0.1) and true gamble probability (across subjects and trials).

Table S5. Model fit statistics for analyses using model-agnostic (MA) and model-based (MB) decision-making measures. Best fits are highlighted in bold. The MA virus likelihood model did not converge and is not reported.

Variable	Model	X^2	CFI	RMSEA	SRMR
Cross-sectional					
All	MA	586.007	0.933	0.057	0.05
All	MB	**610.168**	**0.934**	**0.056**	**0.049**
Longitudinal					
Behavior	MA	**153.208**	0.931	0.077	0.066
Behavior	MB	149.125	**0.943**	**0.07**	**0.063**
General anxiety	MA	327.186	0.916	0.08	0.082
General anxiety	MB	**343.509**	**0.918**	**0.078**	**0.078**
Virus anxiety	MA	97.617	**0.976**	**0.049**	0.058
Virus anxiety	MB	**108.673**	0.974	0.05	**0.057**
Virus likelihood	MA	-	-	-	-
Virus likelihood	MB	191.809	0.956	0.061	0.076
Virus severity	MA	138.662	0.938	0.071	0.068
Virus severity	MB	**141.378**	**0.946**	**0.066**	**0.062**

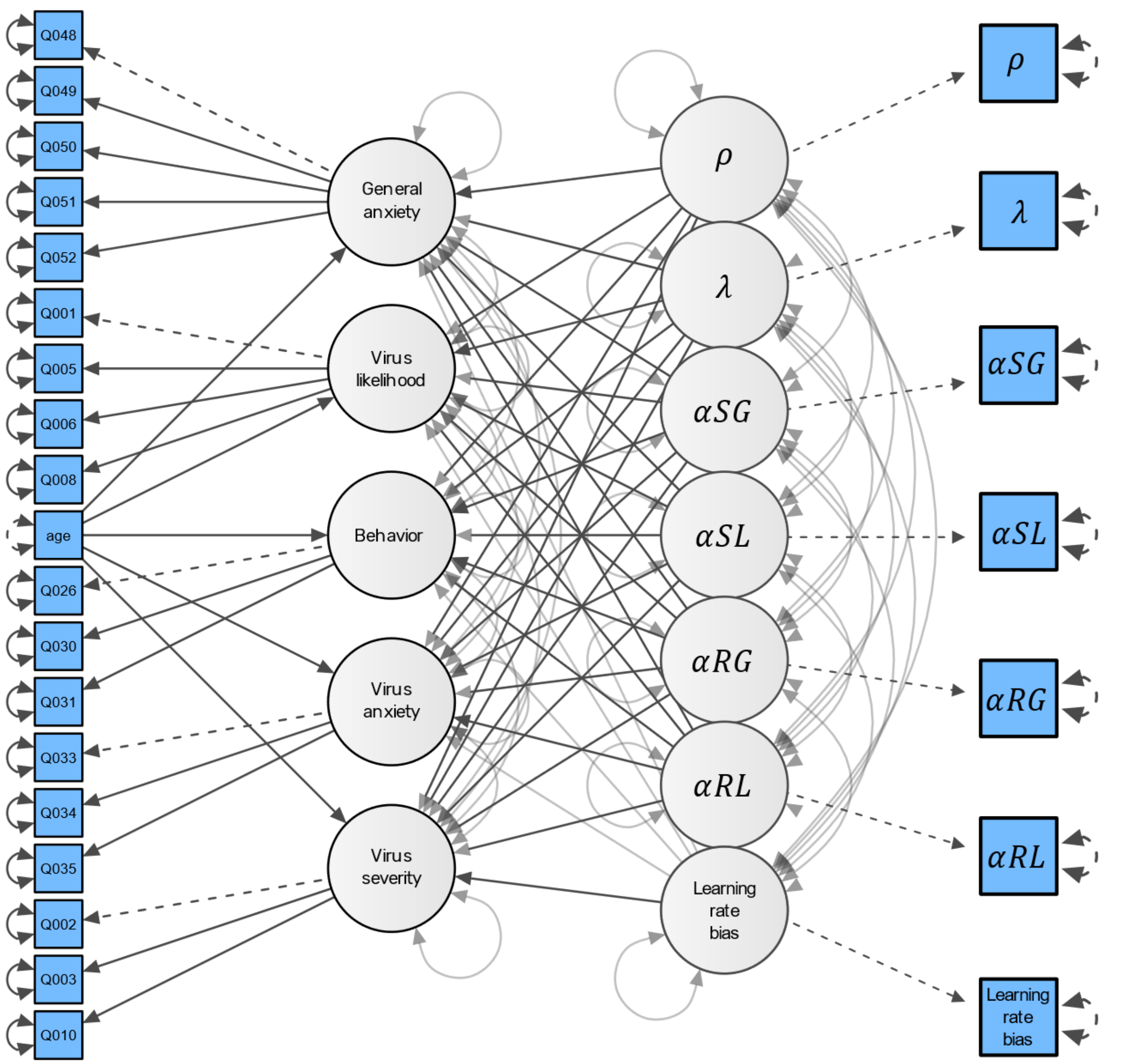

Figure S8. Graphical representation of the latent path model used to assess cross-sectional relationships, which accounts for the factor structure of the self-report models within the model. Variables shown in blue squares represent observed variables, while variables shown in gray circles represent latent variables. Connections between observed and latent variables correspond to factor loadings, while connections between latent variables represent regression paths. Self-connecting paths represent variances, and connections between the same type of latent variable (e.g., between behavioral model parameters) represent covariances. Covariates are omitted from this figure for clarity.

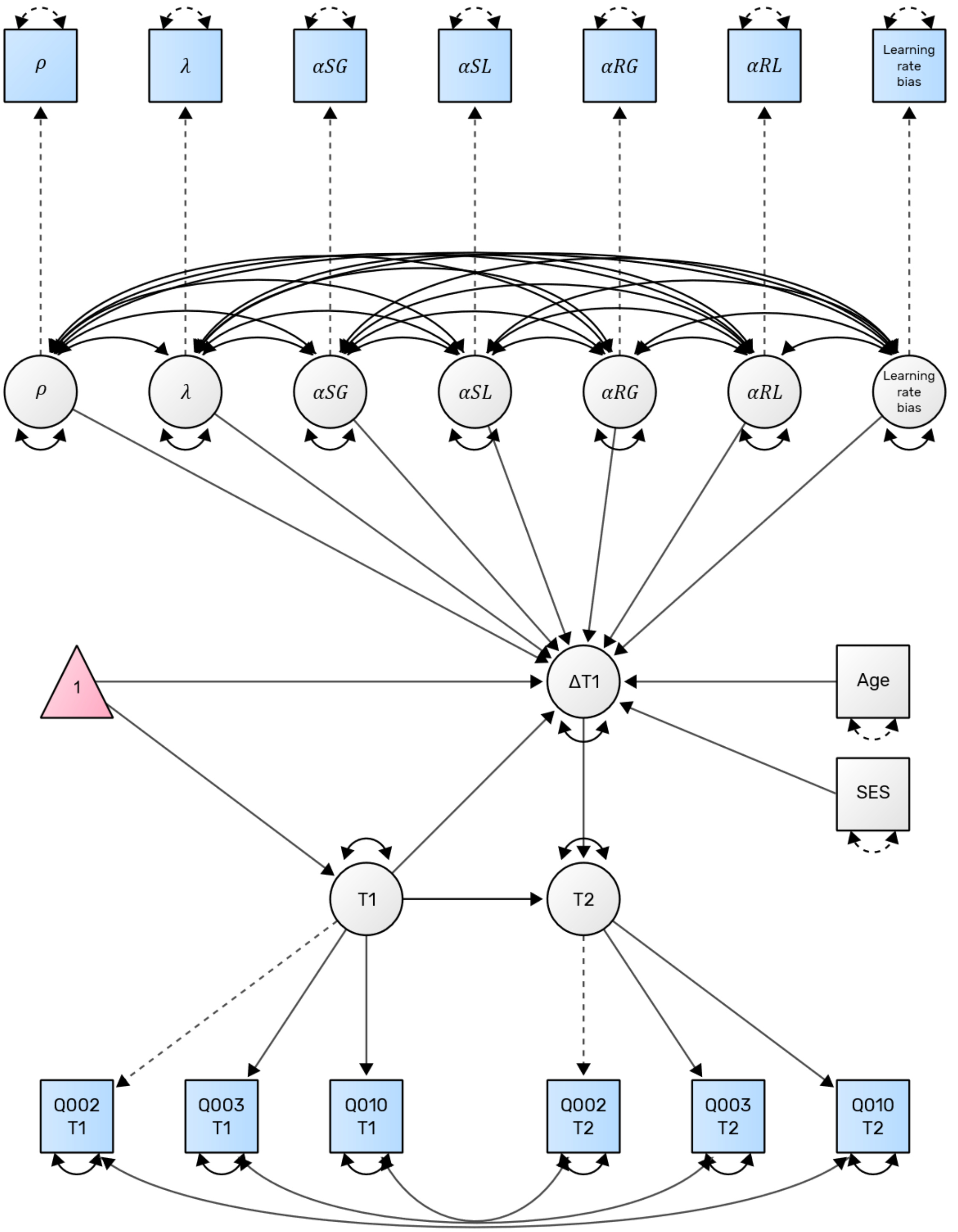

Figure S9. Graphical representation of the latent change score model used to assess longitudinal relationships. Separate models were fit for each of the five factors derived from self-report measures, and this figure shows the model for a single factor (virus severity). T1 represents the T1 factor score, while T2 represents the value of this variable at T2. The change between T1 and T2 is represent by the latent variable ΔT1. The red triangular variable represents an intercept.
